# Mass Media Exposure and Safer Sex Negotiation among Women in Sexual Unions in Sub-Saharan Africa: Analysis of Demographic and Health Survey Data

**DOI:** 10.3390/bs11050063

**Published:** 2021-04-28

**Authors:** Richard Gyan Aboagye, Bright Opoku Ahinkorah, Abdul-Aziz Seidu, Collins Adu, John Elvis Hagan, Hubert Amu, Sanni Yaya

**Affiliations:** 1 School of Public Health, University of Health and Allied Sciences, Ho PMB 31, Ghana; raboagye18@sph.uhas.edu.gh; 2 School of Public Health, Faculty of Health, University of Technology Sydney, Sydney, NSW 2007, Australia; brightahinkorah@gmail.com; 3 Department of Population and Health, University of Cape Coast, Cape Coast PMB TF0494, Ghana; abdul-aziz.seidu@stu.ucc.edu.gh; 4 College of Public Health, Medical and Veterinary Services, James Cook University, Townsville, QLD 4811, Australia; 5 Department of Health Promotion, Education and Disability Studies, Kwame Nkrumah University of Science and Technology, Kumasi PMB, Ghana; collinsadu80@yahoo.com; 6 Department of Health, Physical Education, and Recreation, University of Cape Coast, Cape Coast PMB TF0494, Ghana; 7 Neurocognition and Action-Biomechanics-Research Group, Faculty of Psychology and Sport Sciences, Bielefeld University, Postfach 10 01 31, 33501 Bielefeld, Germany; 8 Department of Population and Behavioural Sciences, School of Public Health, University of Health and Allied Sciences, Ho PMB 31, Ghana; hamu@uhas.edu.gh; 9 School of International Development and Global Studies, University of Ottawa, Ottawa, ON K1N 6N5, Canada; sanni.yaya@uOttawa.ca; 10 The George Institute for Global Health, Imperial College London, London W12 0BZ, UK

**Keywords:** mass media exposure, public health, sexual autonomy, sub-Saharan Africa, women

## Abstract

(1) Background: Improving sexual autonomy among women in sexual unions comes with various benefits, including the reduction of sexually transmitted and blood-borne infections. We examined the relationship between mass media exposure and safer sex negotiation among women in sub-Saharan Africa (SSA). (2) Methods: The study involved a cross-sectional analysis of Demographic and Health Survey (DHS) data of 29 sub-Saharan African countries. A total of 224,647 women aged 15–49 were included in our analyses. We examined the association between mass media exposure and safer sex negotiation using binary logistic regression analysis. The results are presented using a crude odds ratio (cOR) and adjusted odds ratio (aOR), with their respective confidence intervals (CIs). Statistical significance was set at *p* < 0.05. (3) Results: The overall prevalence of safer sex negotiation among women in sexual unions in SSA was 71.6% (71.4–71.8). Women exposed to mass media had higher odds of negotiating for safer sex compared with those who had no exposure (aOR = 1.94; 95% CI = 1.86–2.02), and this persisted after controlling for covariates (maternal age, wealth index, maternal educational level, partner’s age, partner’s educational level, sex of household head, religion, place of residence, and marital status) (aOR = 1.40; 95% CI = 1.35–1.46). The disaggregated results showed higher odds of safer sex negotiation among women exposed to mass media in all the individual countries, except Ghana, Comoros, Rwanda, and Namibia. (4) Conclusions: The findings could inform policies (e.g., transformative mass media educational seminars) and interventions (e.g., face-to-face counselling; small group sensitization sessions) in SSA on the crucial role of mass media in increasing safer sex practice among women in sexual unions. To accelerate progress towards the achievement of the Sustainable Development Goal five’s targets on empowering all women and safeguarding their reproductive rights, the study recommends that countries such as Ghana, Comoros, Rwanda, and Namibia need to intensify their efforts (e.g., regular sensitization campaigns) in increasing safer sex negotiation among women to counter power imbalances in sexual behaviour.

## 1. Background

Improving sexual autonomy among women in sexual unions comes with various benefits, which include the reduction of sexually transmitted and blood-borne infections (STBBIs) [1]. Women, in particular, have been disproportionately impacted by sexually transmitted and blood-borne infections, especially HIV/AIDS [2], due to low sexual autonomy from gender inequalities. Sexual autonomy among women in sexual unions is the control women have over their own lives and the extent to which they have an equal voice with their partners in matters affecting themselves [3]. To achieve this objective, the Sustainable Development Goal (SDG) five focuses on women empowerment to advance their rights in reproductive health decision-making, attitudes, and overall ability to negotiate for safer sex from their male partners [4,5]. Pursuant to this goal, policymakers, especially those in low-and-middle-income countries (LMICs), are beginning to pay particular attention to issues relating to women’s sexual autonomy, which includes the ability to ask the partner to use a condom during sex and the ability to refuse the partner sex [6].

In sub-Saharan Africa (SSA), particularly, the normative societal organization is based on a patriarchal system where men exercise power over women [6]. Various religious and cultural traditions and beliefs restrict sexual autonomy among women and place them in subordination to men [6]. Over the past two decades, media has been considered a powerful tool for bringing women’s rights issues to the attention of a wider public [7] and has also been used as an attempt to enhance various health behaviours in mass populations [8]. Some campaigns incorporate new-age media such as the internet, computers, and mobile phones [9,10]. The advent of the new-age media has proven potential for mobilizing attention and accountability to women’s rights, and challenging discrimination against them [11]. A lack of mass media exposure has been identified as one of the critical factors that contribute to the rise in the incidence of STBBIs among women in sexual unions [12,13]. Previous studies have revealed that mass media influences women’s ability to negotiate for safer sex [12,14,15].

In SSA, women’s ability to negotiate for safer sex has been a major challenge associated with STBBIs since men are regarded as more powerful when it comes to sexual decision-making [12,16]. Apart from mass media exposure, studies have shown associations between some socio-demographic, economic, and cultural determinants (such as place of residence, marital status, age, and educational level) and women’s ability to negotiate for safer sex [14,15,17].

Evidence suggests that mass media exposure enhances one’s ability to negotiate for safe sex, which in turn is linked to the reduction in STBBIs such as HIV/AIDS [18]. For instance, a woman who has knowledge on STBBIs such as HIV/AIDS and asks a partner who has contracted or been exposed to STBBIs (HIV/AIDS) to use a condom or refuse the partner sex may have a lower likelihood of being infected with the virus. Despite the established linkage between mass media exposure and safer sex negotiation and how this linkage contributes to a reduction in STBBIs, there is a paucity of empirical literature on this phenomenon in SSA, calling for urgent attention. Particularly, there seems to be limited evidence on the association between mass media exposure and safer sex negotiation among women in sexual unions in the sub-region. We, therefore, examined the prevalence of mass media exposure and safer sex negotiation among women in sexual unions in SSA using Demographic and Health Survey (DHS) data. We also assessed the association between mass media exposure and safer sex negotiation among women in sexual unions in SSA. The findings of the study could help inform policy formulation in the sub-region to reduce the prevalence of STBBIs.

## 2. Materials and Methods

### 2.1. Data Source and Study Design

The study involved a cross-sectional analysis of the DHS data from 29 Sub-Saharan African countries. We used the data from the DHS conducted between 2010 and 2019. The DHS is a nationally representative study conducted in over 85 LMICs [19]. The survey collects data on varied issues ranging from men’s health, maternal and child health, reproductive health, nutrition, and substance use [19]. To ensure consistency in data collection across countries, the DHS uses a standardised questionnaire comparable across countries for data collection, and the questionnaire is often translated into the major local languages of the countries involved. To ensure the validity of the translated questionnaires, the DHS reports that the translated questionnaires together with the version in English were pretested in English and the local dialect. After that the pretest field staff actively discussed the questionnaires and made suggestions to modify all versions. Following field practice, a debriefing session was held with the pretest field staff, and modifications to the questionnaires were made based on lessons drawn from the exercise. Details of the sampling methods, procedures and implementation can be found on the DHS website in each country final report [19,20]. The survey utilized a two-stage cluster sampling technique with the detailed sampling process highlighted in a previous study [20]. Data for this study were extracted from the women’s file. A total of 224,647 women aged 15–49 with complete cases of variables of interest were included in the analyses (Table 1). The dataset is freely available for download at https://dhsprogram.com/data/available-datasets.cfm and was accessed on 8 March 2021. We relied on the “Strengthening the Reporting of Observational Studies in Epidemiology” (STROBE) guideline in writing the manuscript [21]. The survey years, weighted samples and percentages across all the studied countries in SSA are presented in Table 1.

### 2.2. Study Variables

#### 2.2.1. Outcome Variable

Safer sex negotiation was the outcome variable in the present study. This variable was created as an index of two variables (refuse sex and ask for condom use). In the first variable (refuse sex), the respondents were asked “whether they can refuse sex with their partners” whereas the question for the second variable (ask for condom use) was “whether the respondent can ask their partners to use a condom”. The two variables had the same response options (1 = no; 2 = yes; and 3 = don’t know/not sure/depends). In the present study, the women whose response option was 3 = don’t know/not sure/depends were dropped. Later, safer sex negotiation was created using the remaining two responses. The women who responded “yes” in at least one of the two variables were said to have safer sex negotiations. Those that responded “no” in both variables were categorized as not having safer sex negotiation. This categorization was informed by previous studies that used the DHS dataset [22,23,24].

#### 2.2.2. Key Explanatory Variable

The key explanatory variable was mass media exposure. Mass media was generated from three variables (frequency of reading newspaper/magazine, listening to the radio, and watching television). All three variables had the same response options (0 = not at all; 1 = less than once a week; 2 = at least once a week; and 3 = almost every day). The response options in each of the three variables were recoded into “no” (not at all) and “yes” (less than once a week, at least once a week, and almost every day). Several studies using the DHS dataset have used the same categorization in assessing diverse health and social outcomes [7,25]. 

#### 2.2.3. Covariates

The study controlled for nine variables in determining the association between mass media exposure and safer sex negotiation. These variables include maternal age, wealth index, maternal educational level, partner’s age, partner’s educational level, sex of household head, religion, place of residence, and marital status. The covariates used were selected based on their availability in the dataset and parsimony with safer sex negotiation from the literature [22,23,24,26,27,28]. The study used the existing coding of maternal age, wealth index, sex of household, and residence found in the standard DHS. The husband/partner’s age was recoded as 15–19; 20–24; 25–29; 30–34; 35–39; 40–44; and 45 years and above. Educational level was coded as no education, primary, secondary or higher for the women and their partners. Marital status was recoded as married and cohabiting. Religious affiliation was coded as Christianity, Islam, African Traditional, no religion, and others.

### 2.3. Statistical Analyses

Data extraction, recoding, and final analyses were carried out using Stata version 16.0 (Stata Corporation, College Station, TX, USA). Three levels of analyses were conducted in this study. In the first analysis, percentages with confidence intervals were used to present the results of the prevalence of safer sex negotiation and mass media exposure (Table 2). Secondly, the Pearson chi-square test of independence was conducted to determine the distribution and relationship between safer sex negotiation and mass media across the various countries. Later, two binary logistic regression models were built to determine the effect of mass media exposure on safer sex negotiation in all the 26 countries. The first model (Model I) examined the association between mass media exposure alone and safer sex negotiation. The second model (Model II) was built to determine the association between mass media exposure and safer sex negotiation, while adjusting for the covariates. Results on the association between mass media exposure and safer sex negotiation was disaggregated for each of the countries considered in the study to understand the country-specific variations in the association using two binary logistic regression models. The results of the regression analyses were presented in a tabular form using crude odds ratio (cOR) and adjusted odds ratio (aOR) with their respective confidence intervals (CIs). Statistical significance was set at *p* < 0.05 in the chi-square and regression analysis. The women’s sample weights (v005/1,000,000) were applied to obtain unbiased estimates according to the DHS guidelines and the survey command (SVY) in Stata was used to adjust for the complex sampling structure of the data in the chi-square and regression analyses. Additionally, to check for the existence of multicollinearity among the variables used, a multicollinearity test was conducted using the variance inflation factor (VIF). The results showed a mean VIF of 2.54. Hence, there was no evidence of multicollinearity among the variables studied.

### 2.4. Ethical Approval

Ethical permissions were not required for this study since the DHS datasets, which are publicly available, were used. The DHS reports showed that ethical clearances were obtained from the Ethics Committee of ORC Macro Inc. as well as the Ethics Boards of partner organizations of the various countries such as the Ministries of Health. The survey was conducted with adherence to the standards for ensuring the protection of respondents’ privacy. ICF International ensures that the survey complies with the U.S. Department of Health and Human Services’ regulations for the respect of human subjects. During each of the surveys, the women, including those below 16 years, provided either written or verbal consent prior to the data collection. Further information about the DHS data usage and ethical standards is available at http://goo.gl/ny8T6X and was accessed on 8 March 2021.

## 3. Results

### 3.1. Prevalence of Mass Media Exposure and Safer Sex Negotiation

The prevalence of safer sex negotiation among women in SSA was 71.6% (71.4–71.8). Women in Namibia had the highest prevalence (98.4% [97.8–98.8]) while those from Mali had the lowest (39.6% [36.9–42.4]). The proportion of women exposed to mass media was 67.6% (67.4–67.8), with Gabon having the highest (95.9% [94.8–96.7]) and Chad the lowest (30.6% [27.0–34.4]) (Table 2).

### 3.2. Distribution of Safer Sex Negotiation across Mass Media Exposure

In all the countries studied, we found significant positive association between mass media exposure and safer sex negotiation. Specifically, safer sex negotiation was higher among women who were exposed to media (76.1%), compared with those not exposed (62.2%). Country-specific results on the positive association between mass media exposure and safer sex negotiation were found in all the countries, except Comoros and Rwanda (Table 3).

### 3.3. Multivariable Logistic Regression Analysis on Mass Media Exposure and Safer Sex Negotiation among Women in SSA

Findings from the logistic regression analysis of the association between mass media exposure and safer sex negotiation are presented in Table 3. In all the countries considered in this study, women who were exposed to mass media had higher odds of negotiating for safer sex compared with those who had no exposure (aOR = 1.94; 95% CI = 1.86–2.02), and this persisted after controlling for covariates (maternal age, wealth index, maternal educational level, partner’s age, partner’s educational level, sex of household head, religion, place of residence, and marital status) (aOR = 1.40; 95% CI = 1.35–1.46). Other covariates that showed positive associations with safer sex negotiation were maternal age, cohabiting status, maternal educational level, paternal educational level, female household head, richer, and richest wealth index. Women whose religious affiliations were Islam (aOR = 0.43; 95% CI = 0.41–0.45), African Traditional (aOR = 0.75; 95% CI = 0.66–0.85), and no religion (aOR = 0.72; 95% CI = 0.65–0.81) had lower odds of negotiating for safer sex. Additionally, women residing in rural areas were less likely to negotiate for safe sex (aOR = 0.87; 95% CI = 0.82–0.92) (Table 4). The disaggregated results showed higher odds of safer sex negotiation among women exposed to mass media in all the individual countries, except Ghana, Comoros, Rwanda, and Namibia (Table 5).

## 4. Discussion

Women’s sexual autonomy is important, not only for human rights purposes but other wellbeing consequences such as reproductive health and holistic wellbeing [29]. Similarly, the importance of mass media in behaviour change agenda such as promoting reproductive health among women is of no doubt [7]. We examined the association between mass media exposure and safer sex negotiation among women in sexual unions in SSA. Our findings show that the prevalence of safer sex negotiation among women in SSA was high (71.6%), with women in Namibia having the highest prevalence (98.4%) and Mali, the lowest (39.6%). The proportion of women exposed to mass media was 67.6%, with Gabon having the highest prevalence (95.9%) and Chad, the lowest prevalence (30.6%). We also found a significant positive association between mass media exposure and safer sex negotiation among women in sexual unions in SSA. Specifically, women who were exposed to mass media had higher odds of negotiating for safer sex.

We found a 71.6% prevalence of safer sex negotiation among women in sexual unions in SSA. The high prevalence of safer sex negotiation found in this study corroborates the findings of previous studies that found the prevalence of safer sex negotiation to be 77.1% [30] and 83.4% [31] respectively. There were country level variations in the prevalence of safer sex negotiation among women in sexual unions in SSA. While Namibia had the highest (98.4%) safer sex negotiation, Mali recorded the least (39.6%) safer sex negotiation among women. A possible explanation for this finding could be that compared to Mali, women in Namibia have been exposed to mass media to make decisions regarding their sexual life. The finding suggests that countries in SSA are still deprived of the necessities such as mass media campaigns that lead to improved sexual autonomy among women. This outcome has negative implications for the achievement of the Sustainable Development Goal (SDG) Five targets on empowering all women and safeguarding their reproductive rights by the year 2030 [4]. Therefore, policies and interventions that promote the reproductive health of women regarding their sexuality in SSA are in the right direction in the 21st century, where gender equality is mostly advocated [5].

Although the prevalence of safer sex negotiation is high amidst widespread reports of unequal sexual power relations, gender inequalities, and socio-cultural barriers to safer sex negotiation among women in sexual unions in SSA [32,33], this study showed a high proportion of mass media exposure among women in sexual unions in SSA. The high prevalence of safer sex negotiation in this study is, however, consistent with a previous study conducted in Ethiopia [18]. This could be attributed to the educational level of the respondents, as the majority of the respondents had at least completed primary education. Women who are educated are more likely to be able to negotiate for safer sex compared to their counterparts who are not educated [18].

Current results showed that women who are exposed to media had higher odds of negotiating for safer sex compared to women who were not exposed to media. The findings are in line with the findings of previous studies that have found that mass media exposure enhances women’s autonomy [7,9,11,34], and this includes the ability to negotiate for safer sex. The findings possibly suggest that, generally, mass media is a great avenue for negotiating for safer sex, as some literature has explained [9,34]. However, caution needs to be taken about the specific media to be used to reach out to the particular women of interest. 

In terms of country-specific variations, there were higher odds of safer sex among women exposed to mass media in all the individual countries, except Ghana, Comoros, Rwanda, and Namibia. This finding corroborates findings from a previous study in Ethiopia [18]. Mass media is widely reported in the literature to have the ability to influence people’s sexual behaviour and sexual autonomy [10]. It is therefore no surprise that women in sexual unions in SSA who were exposed to mass media were more likely to negotiate for safer sex. The association between mass media exposure and safer sex negotiation implies that women’s empowerment through media exposure enhances their ability to negotiate for safer sex. This is supported by the theory of gender and power [35]. The theory of gender and power is centred on the idea that sexual practices which encompass safer sex negotiation result from consequences of unequal power relations that are structurally embedded in a patriarchal system [26]. Thus, enhancing women’s empowerment through strategies such as mass media exposure reduces such unequal power relations [35].

### 4.1. Strengths and Limitations 

Nationally representative data were employed to assess mass media exposure and safer sex negotiation among women in sexual unions in SSA. The study has offered insights on the importance of mass media in negotiation for safer sex. The wide coverage and rigour of the analytical procedure have enhanced the prospects of generalising the findings to other contexts where safer sex negotiation of women is to be improved. However, due to the cross-sectional nature of the study design, causal inference cannot be drawn from current outcomes. The relationships established between the explanatory and outcome variables may vary over time. Again, the content, intensity, and frequency of mass media exposure were not considered in this study. Moreover, this study could not cater for the interaction between variables. For instance, a better socioeconomic status may be accompanied by better possibilities of accessing media and (probably) a better situation for women. However, such interactions were not considered in this study. Lastly, the time when the surveys were conducted varied by up to nine years across studied countries, and that needs to be considered as it may affect the comparisons due to the time effect.

### 4.2. Policy and Public Health Implications

Safer sex negotiation remains a critical issue in sexual and reproductive health decision-making, especially in SSA, where patriarchal norms (e.g., men culturally conditioned to demand sex) and other socio-cultural barriers (e.g., power distance) facilitate unequal sexual power relations. This study established the varied prevalence of safe sex negotiation and mass media exposure as well as their connection across different sub-Saharan countries among women in sexual unions. Governments and other stakeholders should target regular sexuality education programmes using the mass media on behaviour change strategies (e.g., persuasive communication) to strengthen safe sexual behaviour through culturally appropriate messages. These programmes should be done most especially in countries where mass media exposure has no association with safer sex negotiation (Ghana, Comoros, Rwanda, and Namibia as well as countries with low prevalence in safer sex negotiation and mass media exposure (e.g., Mali, Chad). Creating more exposure for women to talk about safe sex could help minimize existing social norms on their passive role, often associated with limited inter-partner sexual interactions or communication, would be necessary. Media outlets in these countries should have regular sensitization programmes that provide adequate information on behavioural change (e.g., condom use), perhaps in their local dialects, as a preventive strategy against unwanted pregnancy and sexually transmitted infections, including HIV/AIDS. Other interventions should create more opportunities for women to have regular interactions about their sexual lives and the potential implications of not safely negotiating for their sexual engagements. Future research could examine the type of media exposure on safer sex negotiation through longitudinal designs to identify clear patterns over time to guide appropriate reproductive health interventions and policy in SSA countries.

## 5. Conclusions

The overall prevalence of safer sex negotiation among women in sexual unions was relatively high and similar to mass media exposure. The study also showed a strong statistically significant association between mass media exposure and safer sex negotiation. These findings will inform policies (e.g., transformative mass media educational seminars) and programmes (e.g., face-to-face counselling; small group sensitization campaigns) in the SSA region on the crucial role of mass media in increasing safer sex practice among women. To accelerate progress towards the achievement of the SDG Goal five on empowering all women and safeguarding their reproductive rights, the study recommends that countries such as Ghana, Comoros, Rwanda and Namibia need to intensify their efforts in increasing safer sex negotiation among women in sexual unions to counter power imbalances in sexual behaviour.

## Figures and Tables

**Table 1:** Description of Sample

Countries	Year of Survey	Weighted *n*	Weighted %
**Central Africa**			
Angola	2015–2016	6670	2.97
Cameroon	2018	7029	3.13
Chad	2014–2015	2900	1.29
Congo DR	2013–2014	10,850	4.83
Congo	2011–2012	5822	2.59
Gabon	2012	3820	1.70
**West Africa**			
Burkina Faso	2010	13,100	5.83
Benin	2017–2018	4784	2.13
Cote D’lvoire	2011–2012	5503	2.45
Ghana	2014	5007	2.23
Gambia	2013	6243	2.78
Guinea	2018	6225	2.77
Liberia	2013	5124	2.28
Mali	2018	7854	3.50
Nigeria	2018	27,451	12.22
Sierra Leone	2019	9070	4.04
Senegal	2010–2011	9044	4.03
Togo	2013–2014	5677	2.53
**East Africa**			
Burundi	2016–2017	9572	4.26
Comoros	2012	2429	1.08
Ethiopia	2016	9618	4.28
Kenya	2014	8228	3.66
Rwanda	2014–2015	6736	3.00
Uganda	2016	10,621	4.73
**Southern Africa**			
Lesotho	2014	3498	1.56
Malawi	2015–2016	15,695	6.99
Namibia	2013	2785	1.24
Zambia	2018	7324	3.26
Zimbabwe	2015	5968	2.66
All countries		224,647	100.00

**Table 2:** Proportion of women in sexual unions who are exposed to mass media and can negotiate for safer sex negotiation in sub-Saharan Africa.

Countries	Mass Media Exposure	Safer Sex Negotiation
	% (95% CI)	% (95% CI)
All countries	67.6 (67.4–67.8)	71.6 (71.4–71.8)
**Central Africa**		
Angola	74.0 (70.8–76.9)	71.6 (68.0–74.8)
Cameroon	57.3 (52.7–61.9)	74.1 (71.3–76.7)
Chad	30.6 (27.0–34.4)	55.4 (51.9–58.9)
Congo	71.6 (68.4–74.5)	86.0 (84.2–87.6)
Congo DR	44.5 (41.4–47.7)	75.7 (73.6–77.6)
Gabon	95.9 (94.8–96.7)	92.8 (91.3–94.1)
**West Africa**		
Burkina Faso	73.8 (71.8–75.7)	61.3 (59.1–63.5)
Benin	62.2 (59.6–64.8)	62.8 (60.4–65.0)
Cote D’lvoire	65.8 (62.1–69.4)	65.9 (62.7–68.9)
Gambia	90.0 (87.7–91.8)	60.7 (57.4–63.9)
Ghana	92.0 (90.2–93.4)	84.1 (82.0–86.0)
Guinea	66.1 (63.2–69.0)	49.8 (47.4–52.2)
Liberia	74.9 (71.6–77.9)	88.0 (85.8–90.0)
Mali	79.8 (77.6–81.9)	39.6 (36.9–42.4)
Nigeria	63.5 (61.5–65.4)	61.6 (59.8–63.3)
Senegal	89.8 (88.0–91.3)	41.9 (39.3–44.5)
Sierra Leone	47.1 (44.2–49.9)	72.2 (70.1–74.2)
Togo	70.4 (67.6–73.1)	80.8 (78.8–82.07)
**East Africa**		
Burundi	48.0 (46.2–49.8)	77.7 (76.5–79.0)
Comoros	79.3 (76.1–82.1)	66.1 (62.7–69.3)
Ethiopia	38.8 (35.6–42.1)	52.8 (49.6–56.0)
Kenya	86.7 (85.5–87.8)	86.9 (85.8–87.9)
Rwanda	86.4 (85.1–87.6)	93.6 (92.8–94.3)
Uganda	78.3 (76.8–79.7)	92.3 (91.5–93.1)
**Southern Africa**		
Lesotho	81.3 (78.6–83.7)	95.3 (94.4–96.1)
Malawi	57.0 (55.2–58.7)	82.0 (80.9–83.1)
Namibia	90.8 (89.1–92.2)	98.4 (97.8–98.8)
Zambia	61.5 (58.8–64.2)	79.6 (77.8–81.3)
Zimbabwe	75.4 (73.0–77.6)	86.3 (85.1–87.4)

CI = Confidence Interval; Congo DR = Congo Democratic Republic.

**Table 3:** Mass media and refuse sex, condom use, and safer sex negotiation by countries.

Countries	Refuse Sex		*p*-Values	Ask for Condom Use		*p*-Values	Safer Sex Negotiation		*p*-Values
	Not Exposed to Mass Media	Exposed to Mass Media		Not Exposed to Mass Media	Exposed to Mass Media		Not Exposed to Mass Media	Exposed to Mass Media	
All countries	55.1	66.8	<0.001	42.2	61.1	<0.001	62.2	76.1	<0.001
**Central Africa**									
Angola	44.1	73.2	<0.001	34.5	70.5	<0.001	48.7	79.6	<0.001
Cameroon	59.3	78.4	<0.001	30.0	71.0	<0.001	61.1	83.8	<0.001
Chad	44.0	66.5	<0.001	19.3	35.0	<0.001	48.5	71.2	<0.001
Congo	73.0	70.8	0.201	61.7	69.6	0.001	82.8	87.2	0.005
Congo DR	65.0	70.9	<0.001	36.4	50.0	<0.001	72.0	80.3	<0.001
Gabon	79.5	84.0	0.048	65.0	84.5	<0.001	86.4	93.1	<0.001
**West Africa**									
Benin	48.3	64.3	<0.001	33.8	48.4	<0.001	51.6	69.5	<0.001
Burkina Faso	47.8	55.8	<0.001	29.5	41.8	<0.001	53.8	64.0	<0.001
Cote D’lvoire	50.1	62.9	<0.001	28.5	51.6	<0.001	54.3	71.9	<0.001
Gambia	41.0	54.1	0.002	30.5	46.4	<0.001	43.8	62.5	<0.001
Ghana	68.6	76.5	0.006	49.5	71.1	<0.001	73.5	85.0	<0.001
Guinea	36.4	49.4	<0.001	18.7	33.4	<0.001	39.1	55.3	<0.001
Liberia	76.1	88.4	<0.001	43.1	63.2	<0.001	78.8	91.1	<0.001
Mali	21.2	28.1	<0.001	16.4	31.3	<0.001	29.2	42.2	<0.001
Nigeria	42.2	66.0	<0.001	27.3	52.4	<0.001	45.5	70.9	<0.001
Senegal	21.1	30.2	<0.001	16.1	32.1	<0.001	28.6	43.4	<0.001
Sierra Leone	63.6	74.7	<0.001	38.5	54.4	<0.001	66.4	78.8	<0.001
Togo	68.7	77.0	<0.001	53.7	68.1	<0.001	72.9	84.1	<0.001
**East Africa**									
Burundi	58.4	63.3	<0.001	58.3	62.2	<0.001	75.8	79.9	<0.001
Comoros	52.4	53.2	0.819	50.9	56.0	0.160	64.9	66.4	0.597
Ethiopia	39.8	54.8	<0.001	22.7	46.0	<0.001	45.2	64.8	<0.001
Kenya	61.5	78.1	<0.001	56.7	80.1	<0.001	71.0	89.3	<0.001
Rwanda	84.7	83.3	0.299	83.7	84.8	0.431	93.3	93.7	0.640
Uganda	80.6	88.2	<0.001	76.3	82.2	<0.001	87.7	93.6	<0.001
**Southern Africa**									
Lesotho	62.4	74.5	<0.001	86.9	94.4	<0.001	89.8	96.6	<0.001
Malawi	66.9	72.6	<0.001	71.0	78.2	<0.001	78.3	84.8	<0.001
Namibia	88.9	94.9	0.001	88.6	96.4	<0.001	94.8	98.8	<0.001
Zambia	61.3	67.4	<0.001	68.8	75.8	<0.001	75.6	82.1	<0.001
Zimbabwe	67.7	74.2	<0.001	65.5	73.8	<0.001	81.5	87.9	<0.001

Note: Pearson chi-square test was used to obtain *p*-values; percentages are relative to category refuse sex, ask for condom and safer sex negotiation = yes.

**Table 4:** Binary logistic regression analysis of mass media, covariates, and safer sex negotiation.

Variables	Model I cOR (95% CI)	Model II aOR (95% CI)
**Mass media**		
No	1.0	1.0
Yes	1.94 *** (1.86, 2.02)	1.40 *** (1.35, 1.46)
**Maternal age**		
15–19	1.0	1.0
20–24	1.47 *** (1.40, 1.55)	1.30 *** (1.23, 1.38)
25–29	1.51 *** (1.43, 1.58)	1.44 *** (1.35, 1.54)
30–34	1.49 *** (1.42, 1.57)	1.58 *** (1.48, 1.70)
35–39	1.42 *** (1.35, 1.50)	1.69 *** (1.57, 1.82)
40–44	1.31 *** (1.24, 1.39)	1.69 *** (1.56, 1.83)
45–49	1.15 *** (1.08, 1.22)	1.60 *** (1.47, 1.73)
**Marital status**		
Married	1.0	1.0
Cohabiting	2.46 *** (2.31, 2.62)	1.40 *** (1.32, 1.49)
**Maternal educational level**		
No education	1.0	1.0
Primary	3.08 *** (2.97, 3.21)	1.60 *** (1.54, 1.66)
Secondary/higher	5.30 *** (5.05, 5.56)	2.17 *** (2.07, 2.27)
**Religion**		
Christianity	1.0	1.0
Islam	0.25 *** (0.24, 0.26)	0.43 *** (0.41, 0.45)
African Traditional	0.54 *** (0.47, 0.62)	0.75 *** (0.66, 0.85)
No religion	0.50 *** (0.44, 0.56)	0.72 *** (0.65, 0.81)
Others	1.11 (0.86, 1.43)	1.02 (0.82, 1.28)
**Partner age**		
15–19	1.0	1.0
20–24	1.46 ** (1.16, 1.85)	1.25 (0.98, 1.58)
25–29	1.39 ** (1.11, 1.74)	1.12 (0.89, 1.42)
30–34	1.34 * (1.06, 1.68)	1.05 (0.83, 1.33)
35–39	1.24 (0.99, 1.56)	0.96 (0.75, 1.22)
40–44	1.13 (0.90, 1.42)	0.90 (0.71, 1.14)
45 and above	0.94 (0.67, 1.05)	0.80 (0.63, 1.02)
**Partner educational level**		
No education	1.0	1.0
Primary	3.02 *** (2.89, 3.15)	1.42 *** (1.36, 1.48)
Secondary	4.40 *** (4.20, 4.60)	1.48 *** (1.42, 1.55)
**Sex of household head**		
Male	1.0	1.0
Female	1.41 *** (1.35, 1.47)	1.17 *** (1.12, 1.22)
**Wealth index**		
Poorest	1.0	1.0
Poorer	1.18 *** (1.12, 1.23)	1.00 (0.96, 1.05)
Middle	1.44 *** (1.37, 1.52)	1.05 (0.99, 1.10)
Richer	1.86 *** (1.76, 1.98)	1.09 ** (1.03, 1.16)
Richest	2.92 *** (2.73, 3.13)	1.22 *** (1.14, 1.32)
**Place of residence**		
Urban	1.0	1.0
Rural	0.51*** (0.48, 0.54)	0.87*** (0.82, 0.92)

cOR = Crude Odds Ratio; aOR =Adjusted Odds Ratio; * *p* < 0.05 ** *p* < 0.01 *** *p* < 0.001.

**Table 5:** Logistic regression on the association between mass media exposure and safer sex negotiation in sub-Saharan Africa disaggregated by country.

Countries	Model I	Model II
	cOR (95%CI)	aOR (95%CI)
**Central Africa**		
Angola	3.15 *** (2.82, 3.52)	1.33 *** (1.16, 1.52)
Cameroon	2.99 *** (2.67, 3.45)	1.43 *** (1.22, 1.67)
Chad	2.60 *** (2.20, 3.09)	1.72 *** (1.39, 2.13)
Congo	1.57 *** (1.35, 1.82)	1.57 *** (1.31, 1.87)
Congo DR	1.75 *** (1.60, 1.92)	1.34 *** (1.21, 1.49)
Gabon	2.23 *** (1.67, 2.96)	1.77 ** (1.25, 2.49)
**West Africa**		
Burkina Faso	1.52 *** (1.41, 1.65)	1.31 *** (1.20, 1.42)
Benin	2.17 *** (1.92, 2.45)	1.68 *** (1.47, 1.92)
Cote D’lvoire	2.22 *** (1.98, 2.49)	1.23 ** (1.07, 1.41)
Gambia	2.44 *** (2.09, 2.86)	1.82 *** (1.54, 2.14)
Ghana	1.67 *** (135, 2.08)	1.21 (0.96, 1.53)
Guinea	1.95 *** (1.75, 2.17)	1.59 *** (1.42, 1.79)
Liberia	2.76 *** (2.35, 3.24)	2.29 *** (1.92, 2.73)
Mali	1.69 *** (1.50, 1.90)	1.54 *** (1.36, 1.75)
Nigeria	2.84 *** (2.70, 2.99)	1.18 *** (1.11, 1.26)
Senegal	1.60 *** (1.40, 1.82)	1.19 * (1.03, 1.36)
Sierra Leone	2.09 *** (1.89, 2.30)	1.44 *** (1.30, 1.61)
Togo	2.14 *** (1.88, 2.43)	1.47 *** (1.27, 1.69)
**East Africa**		
Burundi	1.33 *** (1.21, 1.47)	1.14 * (1.02, 1.27)
Comoros	1.38 ** (1.13, 1.68)	0.94 (0.74, 1.18)
Ethiopia	2.64 *** (2.42, 2.89)	1.45 *** (1.30, 1.62)
Kenya	4.88 *** (4.32, 5.52)	1.78 *** (1.51, 2.11)
Rwanda	1.11 (0.83, 1.50)	0.94 (0.70, 1.27)
Uganda	1.86 *** (1.60, 2.15)	1.40 *** (1.20, 1.63)
**Southern Africa**		
Lesotho	3.17 *** (2.34, 4.29)	1.84 ** (1.27, 2.66)
Malawi	1.49 *** 1.37, 1.62)	1.23 *** (1.12, 1.35)
Namibia	3.29 *** (1.83, 5.91)	1.10 (0.55, 2.22)
Zambia	1.52 *** (1.35, 1.70)	1.17 * (1.03, 1.33)
Zimbabwe	1.79 *** (1.52, 2.11)	1.46 *** (1.22, 1.76)

Model I: unadjusted model examining the independent association between mass media exposure and safer sex negotiation; Model II: adjusted for maternal age, wealth index, maternal educational level, partner’s age, partner’s educational, sex of household head, religion, residence, and marital status; cOR is the odds ratio, aOR is the adjusted odds ratio. Reference categories were no exposure to mass media; * *p* < 0.05 ** *p* < 0.01 *** *p* < 0.001.

## Data Availability

Dataset and ethical guidelines are publicly available via this link: http://goo.gl/ny8T6X (accessed on 8 March 2021).
